# Self-organising map clustering identifies high-risk clusters of post-acute mortality in a prospective multicentre study of community-acquired pneumonia

**DOI:** 10.1183/23120541.00374-2025

**Published:** 2026-01-19

**Authors:** Hendrik Pott, Swetlana Gaffron, Roman Martin, Dieter Maier, Max Kutzinski, Barbara Weckler, Wilhelm Bertrams, Anna Lena Jung, Katrin Laakmann, Dominik Heider, Claus F. Vogelmeier, Gernot Rohde, the CAPNETZ Study Group, Bernd Schmeck

**Affiliations:** 1 Department of Medicine, Pulmonary and Critical Care Medicine, Clinic for Airway Infections, University Medical Centre Marburg, Philipps-University Marburg, Marburg, Germany; 2 Institute for Lung Research, Universities of Giessen and Marburg Lung Centre, Philipps-University Marburg, Marburg, Germany; 3 Viscovery Software GmbH, Vienna, Austria; 4 Institute of Medical Informatics, University of Münster, Münster, Germany; 5 Labvantage-Biomax GmbH, München, Germany; 6 Member of the German Centre for Lung Research (DZL); 7 Department of Medicine, Pulmonary and Critical Care Medicine, University Medical Centre Marburg, Philipps-University Marburg, Marburg, Germany; 8 CAPNETZ STIFTUNG, Hannover, Germany; 9 Member of the German Centre of Infectious Disease Research, Marburg, Germany; 10 Institute for Lung Health, Giessen, Germany

## Abstract

**Background:**

Community-acquired pneumonia (CAP) is a leading cause of morbidity and mortality. While tools predicting short-term prognosis exist, there is urgent need for the early identification of patients requiring close follow-up monitoring for post-acute mortality. We therefore conducted cluster analysis of baseline clinical data to investigate predictors of post-acute mortality in CAP.

**Methods:**

We analysed 7840 participants from the German CAPNETZ cohort, using self-organising map (SOM)-clustering and survival analyses. Random survival forest (RSF) models were used to identify key predictors of mortality, which were then analysed using time-dependent area under the curve and Cox proportional hazard regression models.

**Results:**

SOM-clustering based on 10 predictors identified 879 (12%, in four clusters) patients with high risk for post-acute (30–180 days) mortality. Across the cohort, age and urea were the most important predictors of post-acute mortality, while in the high-risk cohort, body mass index emerged as the strongest predictor, as identified by RSF modelling. In one high-risk cluster, there was an association with elevated platelet counts (HR: 1.13, 95% CI 1.03–1.21, p=0.01; increments of 40 platelets·nL^−1^, c14, 35% of high-risk patients), in another (c15, 50% of high-risk patients) with elevated urea (HR: 1.06, 95% CI 1.01–1.11, p=0.02) and C-reactive protein (CRP) (HR: 1.27, 95% CI 1.01–1.58, p=0.04).

**Conclusion:**

Using 10 clinical predictors for post-acute mortality in CAP, predictive SOM-clustering revealed several high-risk subgroups, with heterogeneous biomarkers, suggestive of differences in the underlying pathophysiology (thrombocytes, urea, CRP). Adapting medical therapy to these high-risk subgroups may reduce post-acute mortality following CAP.

## Shareable abstract

**Cluster analysis in ∼8000 patients from the CAPNETZ cohort suggests the existence of different high-risk phenotypes for post-acute mortality, with distinctive biomarkers (thrombocytes, urea, CRP)** https://bit.ly/4nIJpQv

## Graphical abstract

**Figure:**
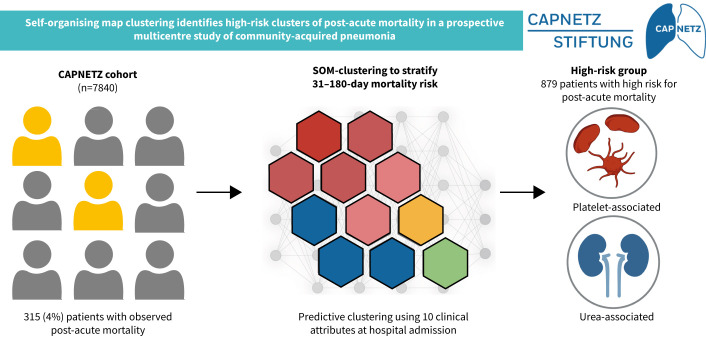
Self-organising map clustering identifies high-risk clusters of post-acute mortality in a prospective multicentre study of community-acquired pneumonia. SOM: self-organising map.

## Introduction

Community-acquired pneumonia (CAP) remains a high mortality disease and global health problem [1]. Over the last decades, evidence has emerged that CAP has a long-lasting impact on individual health, associated with increased mortality [2–5]. While clinicians routinely utilise scores to assess 30-day mortality risk [6, 7], an effective score to guide post-discharge monitoring remains elusive [8].

Several studies have investigated risk factors for post-acute mortality in CAP [3–5, 9–13], including age [4, 12, 14], sex [3, 10, 11] and comorbidities, including cardiovascular [9, 12, 14], pulmonary [12, 14] or neurodegenerative disease [12, 13]. Simultaneously, the first 6 months following CAP are particularly critical, as the majority of deaths occur during this timeframe [12]. However, mortality varies across that period, suggesting the existence of different at-risk groups or phenotypes [15].

Suitable biomarkers for post-acute mortality would enable identification of high-risk patients for targeted intervention [16]. However, while pulmonary rehabilitation is the primary post-CAP intervention, recent analyses suggest a limited effect [17], highlighting the need for better risk stratification. Here, machine learning offers powerful tools for clustering CAP patients based on mortality risk [8], although previous studies have focused on either acute [16, 18–21] or intensive care unit (ICU)-related mortality [22]. A prior CAPNETZ cohort analysis assessed early post-discharge mortality [21], but did not employ clustering methods or investigate mortality beyond 30 days. Taken together, we here hypothesised: 1) the existence of multiple high-risk clusters for post-acute mortality; 2) their identifiability using clinical attributes at hospital admission; and 3) their association with different biomarkers for post-acute mortality.

## Methods

### Study design and patients

CAPNETZ (https://capnetz.de/) is a prospective, multicentric study (NCT02139163). Briefly, patients aged at least 18 years old with CAP, defined as presence of a pulmonary infiltrate on chest radiograph plus at least one of fever (≥38.3°C), cough, purulent sputum and positive auscultatory findings, were included. Exclusion criteria were nosocomial infection, immunosuppression or active tuberculosis, and follow-up was standardised for 6 months. Ethical approval was obtained from participating centres, and all patients provided written informed consent.

Number of acute organ dysfunctions was assessed as the sum of the following conditions (0–9): confusion, tachycardia, hypotension, tachypnoea, dyspnoea, pleuritic pain, oxygen application, breathing invasively, low O_2_ saturation. The number of comorbidities (0–12) was defined as the sum of the following diagnoses: chronic symptoms, malignancy, chronic respiratory disease, heart failure, other heart disease, chronic liver disease, chronic renal disease, cerebrovascular disease, other neurological disease, diabetes mellitus, enteral tube feeding and chronic invasive ventilation *via* tracheostomy.

Patients admitted between 2002 and 2017, with a follow-up of up to 180 days after hospital admission, were available in our dataset. Patient information comprised >150 variables, encompassing clinical and biomarker data, such as leukocytes and platelets; radiological information, such as multilobar infiltrates and pleural effusion; vaccination status; medication, such as treatment with inhaled corticosteroids; and prior antibiotic therapy. Blood samples were taken within 48 h of hospital admission. As biomarkers of post-acute mortality might diverge significantly in these populations, we excluded non-hospitalised study participants and nursing home residents from our analysis. From 11 832 available records, 7840 were curated for predictive modelling, and (excluding records missing more than two predictive attributes) 7309 were included in the final clustering (supplementary table S1, supplementary figure S1).

### Generation of cluster models and statistical analysis

Data analysis was conducted using Viscovery SOMine 8.0 by Viscovery Software GmbH (www.viscovery.net; Vienna, Austria). The software applied a nonparametric regression technique, referred to as a self-organising map (SOM), converting multidimensional clinical data into a two-dimensional “map”, in which patient placement was dictated by the respective similarity of their data. In the resulting SOM, proximity indicates higher data similarity. Further detail as to the statistical clustering method implemented is given in the supplementary material.

Preliminary clustering was performed by allocating clustering weight mainly to patient outcome, defined as 30 days mortality, 31–180 days mortality (post-acute mortality) and survival with ICU admission, the necessity to adapt antibiotic treatment or none of the priorly stated. It needs to be mentioned that the clustering process followed a prespecified sequence, starting with the allocation of patients to the mortality clusters and allocating the remaining patients depending on their clinical course. This means that being allocated to a mortality cluster could also involve ICU admission and change of antibiotic treatment.

Using differentially distributed baseline attributes in the preliminary clustering, 10 attributes were selected for predictive clustering, aiming to reproduce patient segregation by outcome.

Descriptive statistical analysis was performed using analysis of variance (ANOVA), Chi-squared and Kruskal–Wallis tests, *post hoc* tests being Games–Howell/Tukey-, Dunn- and Bonferroni-adjusted Chi-squared tests, for continuous, categorical and discrete variables, respectively. Survival analysis was conducted using Kaplan–Meier and Nelson–Aalen models, and log rank tests, to compare clusters and cluster groups. Predictive strength over time was assessed by calculation of the cumulative and dynamic area under the curve (AUC), using stratified cross-fold (5-fold) validation. Effect size of covariates and interactions with cluster membership were tested using Cox proportional hazard regression models and continuous covariates were normalised to enable comparability of coefficients.

Random survival forest (RSF) models were generated on a 0.65:0.35 stratified train-test split, using stratified cross-fold (5-fold) validation, and grid-based hyperparameter search, optimising for concordance index. Datasets for machine-learning analyses consisted of the 55 most important clinical variables. *Post hoc* feature importance analysis was conducted by inspection of permutation-based feature importances. Descriptive statistics were performed in R; all other analyses were conducted using Python (v.3.8.8) with the packages statsmodels v. 0.14 [23], lifelines v.0.27 [24], scikit-learn v.1.3 [25] and scikit-survival v.0.22 [26].

## Results

Data for 11 832 patients from CAPNETZ were available, with 7840 patients being eligible for our analysis (supplementary table S1, supplementary figure S1). Post-acute mortality occurred in 315 (4%), while 280 (3.6%) died within 30 days (table 1, supplementary figures S1–S3). Among survivors, 120 (1.5%) required ICU admission, while 893 (11%) required changes to the initially chosen antibiotic treatment.

**TABLE 1:** Clinical attributes at admission for outcome groups following community-acquired pneumonia

Attribute	D30	D31–180	ICU	Change	Rest	p-value^#^
**Patients, n**	280 (3.6%)	315 (4.0%)	120 (1.5%)	893 (11%)	6232 (79%)	
**Age years**	73.9±12.3	73.6±11.1	63.6±15.8	62.4±17.1	61.2±17.6	<0.001
**Body mass index kg·m^−2^**	24.8±6.2	25.2±5.3	26.6±6.0	25.9±5.4	26.3±5.7	<0.001
**Urea mmol·L^−1^**	12.0±8.0	9.9±8.2	10.0±6.6	7.2±5.3	6.7±4.8	<0.001
**Haemoglobin g·L^−1^**	7.7±1.4	7.8±1.3	8.4±1.4	8.2±1.1	8.3±1.1	<0.001
**C-reactive protein mg·dL^−1^**	152.9±122.1	110.5±97.2	160.9±158.2	157.7±131.6	134.2±121.4	<0.001
**Oxygen saturation %**	89.7±6.8	90.0±7.9	87.8±9.7	92.7±5.3	92.4±5.6	<0.001
**Female**	83.0 (29.6%)	82.0 (26.0%)	39.0 (32.5%)	338.0 (37.8%)	2573.0 (41.3%)	<0.001
**Dyspnoea**	255.0 (93.1%)	258.0 (82.4%)	107.0 (89.2%)	652.0 (73.2%)	4547.0 (73.1%)	<0.001
**Systolic blood pressure <100 mmHg**	77.0 (27.8%)	38.0 (12.1%)	21.0 (17.5%)	92.0 (10.4%)	528.0 (8.5%)	<0.001
**Oxygen application (0,1)**	250.0 (89.6%)	241.0 (76.5%)	111.0 (92.5%)	580.0 (65.1%)	3592.0 (57.7%)	<0.001
**Number of acute organ dysfunctions (0–9)**	4.3±1.5	3.8±1.2	4.8±1.5	3.2±1.6	3.2±1.5	<0.001

Data are presented as mean±sd or n (%). D30: 30-day mortality group; D31–180: 31–180-day mortality group; ICU: group requiring intensive care unit admission; Change: group requiring changes to initial antibiotic treatment; Rest: rest of study cohort. Systolic blood pressure <100 mmHg was assessed as low systolic blood pressure. Number of acute organ dysfunctions was assessed as the number of the following conditions, if exhibited by the patient: confusion, high heart rate, low mean blood pressure, high respiratory rate, dyspnoea, pleural pain, oxygen application, breathing noninvasively, low oxygen saturation. ^#^: Welch-ANOVA; Pearson's Chi-squared test; Kruskal–Wallis rank sum test.

### Patients with post-acute mortality exhibit distinct clinical attributes at baseline

Patients who died post-acutely were older (73.6±11.1 years) compared to ICU survivors (63.6±15.8 years, p<0.001) or those requiring antibiotic changes (62.4±17.1 years, p<0.001, supplementary table S2). Clinically, they had lower O_2_ saturation (90.0±7.9%) than non-ICU survivors (Change: 92.7±5.3%, p<0.001, Rest: 92.4±5.6%, p<0.001) and more frequently presented with dyspnoea (Change: 82.4% *versus* 73.2%, p=0.01; Rest: 82.4% *versus* 73.1%, p=0.002). Conversely, baseline C-reactive protein (CRP; 110.5±97.2 mg·dL^−1^) and haemoglobin (7.8±1.3 g·L^−1^) were significantly lower than in survivors without ICU admission or antibiotic treatment change (134.2±121.4 mg·dL^−1^, p<0.001; 8.26±1.11 g·L^−1^, p<0.001, supplementary table S3). Finally, patients with post-acute mortality, compared to non-ICU survivors (Rest), more often exhibited comorbidities, such as heart failure (47.8% *versus* 31.7%, p<0.001), chronic renal disease (26.2% *versus* 15.9%, p<0.001) or cerebrovascular disease (21.3% *versus* 11.7%, p<0.001).

### Key predictors of post-acute mortality

**FIGURE 1:**
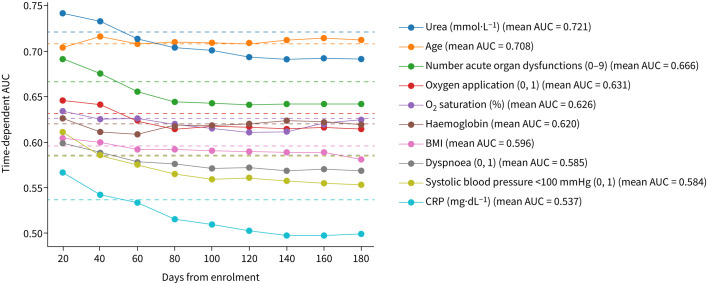
Predictive strength of single baseline attributes over 180-day follow-up. Predictive strength was explored by calculation of cumulative/dynamic area under the curve (AUC), as implemented in the scikit-survival package. In short, the plotted metric illustrates how well a model trained on that attribute can distinguish mortality groups before and after a specific timepoint, for any timepoint of the 180 days follow-up. The mean AUC was indicated by dotted lines in the respective colour used to plot the model. BMI: body mass index; CRP: C-reactive protein.

RSF models identified age and urea as the strongest predictors of mortality during the whole (180 days, Harrel's C 0.81) and post-acute follow-up (30–180 days, Harrel's C 0.79, supplementary tables S4–S5). Time-dependent AUC analysis supported these findings, showing that age and urea consistently had the highest predictive strength over the whole 180-day follow-up period (figure 1). Interestingly, the predictive strength associated with the number of acute organ dysfunctions declined over time, while AUCs for baseline oxygen saturation and haemoglobin remained stable over the whole follow-up period.

### Cluster analysis identifies high-risk groups

**FIGURE 2:**
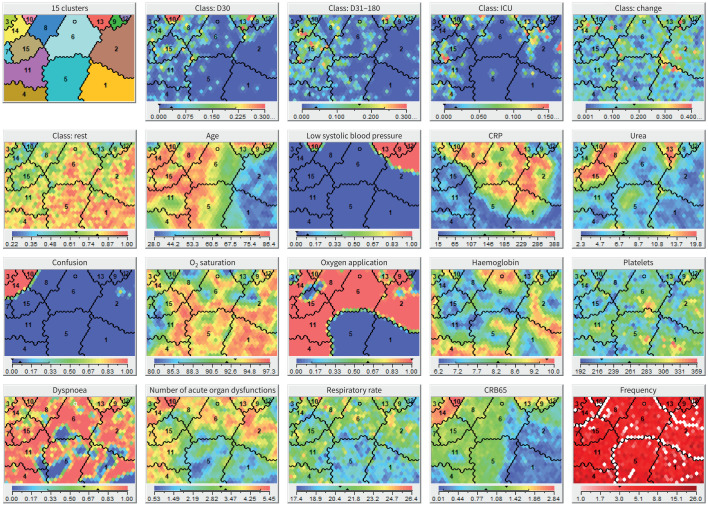
Predictive self-organising map (SOM)-clustering. A predictive SOM-clustering model was generated using the Viscovery software, with the aim of segregating patients by outcome (acute *versus* non-acute mortality, admission with(out) intensive care unit (ICU) or change of antibiotic treatment). Clustering was performed on 7309 patients, based on 10 baseline patient attributes: age, body mass index, dyspnoea, systolic blood pressure <100 mmHg, number of acute organ dysfunctions, oxygen application and (peripheral) saturation, haemoglobin, C-reactive protein (CRP) and urea. Similarity of patient data was encoded by proximity of placement on the map, with more diverse patients at higher distance from each other. Arithmetic mean of assessed values in each node was colour-coded as indicated by the respective panel legends. Cluster numbers were assigned by increasing order of non-acute mortality (in %). D30: 30-day mortality group; D31–180: 31–180-day mortality group; ICU: group requiring ICU admission; Change: group requiring changes to initial antibiotic treatment; Rest: rest of study cohort; CRB65: aggregate score for clinical evaluation of pneumonia severity, consisting of pneumonia-related confusion, respiratory rate >30 breaths·min^−1^, systolic blood pressure <90 mmHg and/or diastolic blood pressure <60 mmHg, and age ≥65 years.

Based on the above results, 10 baseline attributes (age, body mass index (BMI), dyspnoea, systolic blood pressure <100 mmHg, number of acute organ dysfunctions, supplemental oxygen therapy and (peripheral) oxygen saturation, haemoglobin, CRP and urea; table 1) were chosen for clustering analysis, identifying 15 patient clusters (figure 2, table 2). Four clusters (c12–15) exhibited high post-acute mortality (∼10% or higher), while c11 showed moderate mortality rates (6.9%). ICU survivors were concentrated in c7, c8 and c13, while antibiotic-change patients were common in c9, c12 and c14.

**TABLE 2:** Outcomes per cluster in the clustering cohort

Cluster	30-day mortality, n=255 (3.5%)^#^	31–180-day mortality, n=296 (4.0%)^#^	ICU, n=118 (1.6%)^#^	Change of initial antibiotic treatment, n=846 (11.6%)^#^	180-day mortality, n=551 (7.5%)^#^	Overall, n=7309^¶^
**1**	2 (0.2)	7 (0.6)	5 (0.4)	135 (11.1)	9 (0.7)	1216.0 (16.6%)
**2**	13 (1.3)	11 (1.1)	21 (2.0)	142 (13.8)	24 (2.3)	1028.0 (14.1%)
**3**	2 (1.8)	3 (2.7)	1 (0.9)	13 (11.7)	5 (4.5)	111.0 (1.5%)
**4**	15 (2.5)	16 (2.7)	13 (2.2)	57 (9.7)	31 (5.3)	590.0 (8.1%)
**5**	8 (0.7)	35 (3.1)	1 (0.1)	112 (9.9)	43 (3.8)	1129.0 (15.4%)
**6**	29 (3.1)	36 (3.8)	10 (1.1)	126 (13.5)	65 (6.9)	936.0 (12.8%)
**7**	5 (4.0)	5 (4.0)	5 (4.0)	10 (7.9)	10 (7.9)	126.0 (1.7%)
**8**	27 (7.5)	18 (5.0)	12 (3.4)	37 (10.3)	45 (12.6)	358.0 (4.9%)
**9**	8 (7.8)	6 (5.9)	3 (2.9)	19 (18.6)	14 (13.7)	102.0 (1.4%)
**10**	27 (44.3)	4 (6.6)	2 (3.3)	6 (9.8)	31 (50.8)	61.0 (0.8%)
**11**	34 (4.4)	53 (6.9)	16 (2.1)	80 (10.3)	87 (11.3)	773.0 (10.6%)
**12**	4 (9.8)	4 (9.8)	0 (0.0)	7 (17.1)	8 (19.5)	41.0 (0.6%)
**13**	26 (15.6)	17 (10.2)	7 (4.2)	21 (12.6)	43 (25.7)	167.0 (2.3%)
**14**	28 (9.0)	37 (11.9)	12 (3.8)	50 (16.0)	65 (20.8)	312.0 (4.3%)
**15**	27 (7.5)	44 (12.3)	10 (2.8)	31 (8.6)	71 (19.8)	359.0 (4.9%)

Following predictive self-organising map clustering, clinical outcomes were compared between each of the 15 generated clusters. Absolute and relative frequencies were reported. ICU: intensive care unit. ^#^: n (% of patients in cluster); ^¶^: n (% of clustering cohort).

**FIGURE 3:**
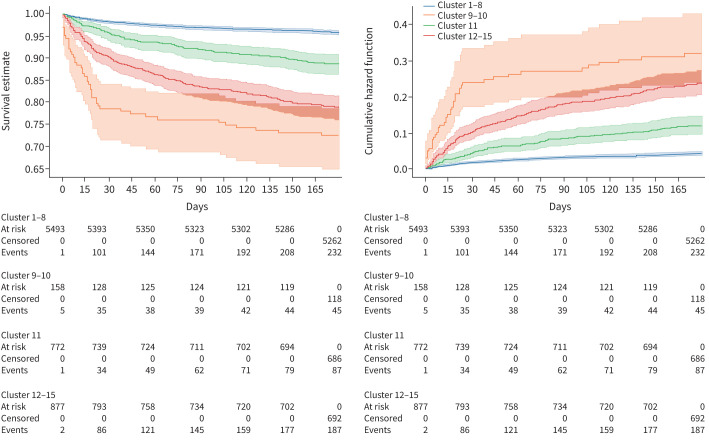
Grouping self-organising map-generated clusters by acute and non-acute mortality. Groups were compared using Kaplan–Meier and Nelson–Aalen models, accompanied by log-rank tests. Clusters 9–10 (c9–10) and c12–15 separated early, with high acute mortality in c9–10, and high non-acute mortality in c12–15, until overlap of confidence intervals following ∼80 days of follow-up. Cluster 11 was regarded as a cluster with intermediate non-acute mortality and therefore not grouped with c1–8. Patients in the dataset were given as “At risk”, patients with mortality were given as “Mortality”. Follow-up was censored at 180 days.

Survival analysis grouped clusters into high- (c12–15), intermediate- (c11) and low-risk (c1–8) cluster groups (figure 3). High-risk clusters differed significantly from low-risk ones (p<0.001), yet mortality differences inside risk groups were minimal (supplementary tables S6–S8). RSF models identified age as the strongest predictor of post-acute mortality in the low-risk groups (Harrel's C: 0.77), while BMI was the strongest predictor in the high-risk group (Harrel's C: 0.66, supplementary tables S4, S9−S10). In the intermediate-risk group, BMI was suggested as the most important feature as well, yet the model did not achieve predictive strength (Harrel's C: 0.48).

### Biomarkers predicting post-acute mortality in high-risk clusters

Investigating for heterogeneity in post-acute mortality pathophysiology, we searched for differences in predictors of post-acute mortality in high-risk clusters. The high-risk group incorporated four clusters (c12–15), encompassing 879 patients, with no significant difference in overall survival between c13, c14 and c15 (supplementary figure S4, supplementary table S8). For c13–15, the most important predictors of overall and non-acute mortality were identified from RSF models (supplementary tables S4, S12 and S13) and then analysed by time-dependent AUC analysis (supplementary figures S5–S7) and Cox modelling (supplementary tables S14–S19). The number of patients in c12 was small, prohibiting a detailed analysis.

In c13 (n=167, 19.0% of high-risk patients), BMI, CRP and leukocyte concentration were the most important predictors of post-acute mortality (supplementary table S12). When comparing the predictive power of these features between c13, c14 and c15, time-dependent AUC analysis highlighted a stronger association of CRP with mortality in c13 (mean AUC: 0.64) over c14 (mean AUC: 0.50) or c15 (mean AUC: 0.55, supplementary figure S5). This was underscored by Cox hazard regression analysis, where interaction modelling showed that CRP was associated with a gradual decrease in post-acute mortality risk for members of c13 (c13×CRP (steps of 40 mg·dL^−1^): 0.79, 95% CI: 0.65–0.96, p=0.02, supplementary table S14). Interestingly, this association was inverted in c15, where CRP, in tendency, was associated with a gradual increase in post-acute mortality risk (c15×CRP: 1.27, 95% CI: 1.01–1.58, p=0.04).

**FIGURE 4:**
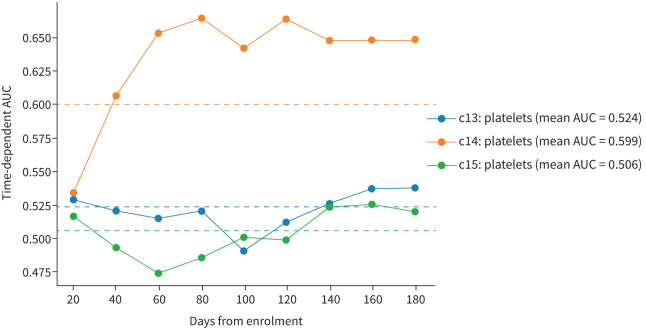
Predictive strength of baseline platelet concentration for 180-day mortality in Clusters 13–15 (c13–15). High post-acute mortality clusters c13–15 were investigated for biomarkers predictive of post-acute mortality, and predictive strength was then explored by calculation of cumulative/dynamic area under the curve (AUC). The mean AUC was indicated by dotted lines in the respective colour used to plot the model.

For c14 (n=312, 35.49% of high-risk patients), heart rate at admission, BMI and platelet concentration were the most important predictors of post-acute mortality. However, while Cox regression analysis highlighted a significant association between heart rate at admission and post-acute mortality in the whole cohort (1.01, 95% CI: 1.00–1.01, p=0.04, supplementary table S15), this association did not differ significantly for clusters 13, 14 or 15. Yet, when inspecting BMI with time-dependent AUC analysis, BMI had the strongest association with post-acute mortality in c14 (0.72, supplementary figure S6), when compared to c13 (mean AUC: 0.64) and c15 (mean AUC 0.64), with Cox regression highlighting a gradual protective effect associated with higher BMI for patients in c14 (0.88, 95% CI: 0.8–0.97, p=0.01, supplementary table S16). Conversely, when investigating baseline platelet concentration, we found a significant association indicating a gradual increase in post-acute mortality risk in the whole cohort (1.03, 95% CI: 1.0–1.06, p=0.03, increments of 40 thrombocytes·nL^−1^, supplementary table S17). Of note, patients in c14 had significantly higher risk of post-acute mortality associated with thrombocyte levels (1.13, 95% CI: 1.03–1.21, p=0.01, increments of 40 thrombocytes·nL^−1^). Simultaneously, in time-dependent AUC analyses, platelets had stronger associations with mortality than in c13 and c15, especially during the post-acute follow-up (figure 4). Interestingly, baseline concentration of platelets did not differ significantly between c13, c14 and c15 (supplementary table S11).

The strongest predictors of post-acute mortality in c15 (n=359, 40.84% of high-risk patients) were BMI, influenza vaccination state and age; although the model's predictive strength was weak (supplementary tables S4, S13). While in c15, post-acute mortality did not display an association with BMI significantly different from the rest of the study cohort, the gradual increase of post-acute mortality with age was significantly lower than in the rest of the study cohort (0.96, 95% CI: 0.92–1.0, p=0.04, supplementary table S18). Conversely, when analysing other blood biomarkers, we found that baseline urea had a stronger association with mortality in c15 (mean AUC: 0.63) than in c13 (0.55) or c14 (0.61, supplementary figure S7), and was associated with significantly higher mortality compared to the rest of the study cohort (1.06 (1.01–1.11), p∼0.02, supplementary table S19).

Overall, these results suggest the existence of various subgroups at high risk for post-acute mortality, with different biomarkers associated with mortality increase, such as urea and CRP (c15) and platelets (c14).

## Discussion

There were three major findings in this study: 1) in CAP, predictive clustering of clinical admission data can discern groups with high, medium and low risk for post-acute mortality; 2) in general, age and urea were the strongest predictors of post-acute mortality, yet BMI was the strongest predictor in the high-risk subgroup; 3) we identified at least two subgroups with high risk for post-acute mortality, one associated with baseline platelet concentration, the other with baseline urea and CRP concentrations.

Analysis of outcome groups yielded a panel of predictive markers, consisting of age, urea, BMI, CRP, dyspnoea, systolic blood pressure <100 mmHg, number of acute organ dysfunctions, oxygen application and (peripheral) saturation, and haemoglobin for predictive clustering. Concordantly, most of these markers have previously been described in association with post-acute mortality [27]. Analysing this panel using time-dependent AUC analysis, we observed a number of predictors with relative stability of predictive strength over follow-up (*e.g.* age, urea and baseline O_2_ saturation), while others declined in predictive strength in the post-acute interval (*e.g.* the number of acute organ dysfunctions and CRP). Interestingly, while CRP is an independent marker of pneumonia severity [28], there exist conflicting reports about its utility as a biomarker for post-acute mortality [11, 29].

Age and urea are long-standing markers of mortality in CAP [14], expectedly ranking as the most important predictors of post-acute mortality in the whole cohort. However, this finding disappeared when inspecting intermediate (c11) and high-risk clusters (c12–15), where age was replaced with (mostly) BMI as the most important predictor of mortality (supplementary tables S9–S10). More so, while age was associated with mortality in the whole cohort, this effect was less pronounced in a subgroup of high-risk patients (Cluster 15, supplementary table S13). Additionally, while previous analyses have highlighted age and low BMI as important risk factors after hospital discharge in CAP [21, 30], our results suggest that subgroups with high risk for post-acute mortality exist, with nuanced responses to these biomarkers, as demonstrated by the associations of age and BMI to clusters 14 and 15. A possible explanation for the heterogeneity observed in effects of age and BMI on post-acute mortality could lie in differing levels of frailty in these clusters [31]. However, as such data were unavailable for our analysis, further research is needed to assess the prevalence of frailty in high-risk subgroups for post-acute mortality following CAP.

The high-risk group for post-acute mortality consisted of four clusters (c12–15), encompassing 879 patients. These clusters represent patients with shared clinical and risk profiles and may guide future post-discharge strategies for patients with CAP. However, one cluster (c12, n=41, 4.6% of high-risk patients) was excluded from further analysis due to lower patient count and was not merged with pre-existing clusters to prevent confounding potential findings.

One subgroup (c14, n=312, accounting for 35% of patients in the high-risk group) exhibited heart rate, BMI and platelet concentration at admission as the most important predictors of post-acute mortality. In this group, we observed protective effects conferred by BMI, while having a gradual increase of mortality risk in association with platelet counts. Curiously, however, baseline platelet concentrations did not differ between high-risk clusters, suggesting that the risk associated with platelets be biologically context-dependent. Another possible explanation may be higher activation grade and hyperaggregation of platelets, which occur in CAP [32]. These findings are in line with two recent trials showing that aspirin reduces hyperaggregation of platelets [33] and 1-year mortality in pneumonia [34]. In this context, one may speculate that the ∼35% of patients found by our clustering approach represent one of the groups benefiting most from platelet inhibition, estimating it at 312 patients. In any case, the results presented here could inform prediction algorithms for further trials of thrombocyte aggregation inhibitors in CAP.

Additionally, we observed at least one other subgroup (c15, ∼41% of high-risk patients) in which baseline BMI, influenza vaccination state and age were the most important predictors of post-acute mortality. Patients in this group had a significantly lower post-acute mortality risk related to age, but higher risk in relation to urea. Additionally, post-acute mortality was associated with higher baseline CRP in this group. Interestingly, previous analyses have highlighted that CRP decline over the hospital stay [35], rather than CRP at admission [36] were associated with post-acute mortality. However, CRP levels at hospital discharge were not available in our dataset.

Several strengths and limitations apply to this study. Analysis was conducted on a large and well-characterised multicentric cohort, yet the longest available follow-up interval was 6 months. As such, this study cannot make a prediction for 1-year mortality, limiting comparability to studies with longer follow-up. Utilisation of RSF models enabled modelling of complex covariate interactions not possible with conventional Cox hazard regression models, yet detailed analysis of patient mortality causes (*e.g.* in a competing risk analysis) was not possible due to lack of data granularity. Last, predictive SOM-clustering allowed for predictions robust to missing values, yet some variables (*e.g.* for comorbidity status) had a high percentage of missing values. Additionally, the identification of optimal clustering conditions, comparing SOM to other machine learning approaches, was beyond the scope of this analysis. It is therefore important to acknowledge that the results are exploratory and require validation in other cohorts.

Our findings have potential clinical implications as early identification of patients in high-risk clusters for post-acute mortality may help guide future post-discharge strategies, for instance by prioritising follow-up for patients with renal risk or thrombotic risk profiles or to select patients benefiting from antiplatelet therapy.

In conclusion, we report the first (SOM-) cluster analysis of post-acute mortality in a multicentric cohort of CAP, showing that baseline attributes can predict multiple subgroups at risk for post-acute mortality. Two high-risk subgroups emerged from our analysis, one (35%) with increased mortality risk associated with platelets, the other (41%) with urea at admission. Further prospective studies will need to explore the necessary treatment interventions for the phenotypes, aiming to reduce associated long-term mortality.

## Data Availability

Data and biomaterials from the CAPNETZ study can be requested by a formal application and will be decided upon by the board of the CAPNETZ STIFTUNG (https://capnetz.de/en/project-application/).

## Supplementary material

**Please note:** supplementary material is not edited by the Editorial Office, and is uploaded as it has been supplied by the author. Supplementary material 00374-2025.SUPPLEMENT FIGURE S1 Patient records available for our analysis 00374-2025.SUPPLEMENT FIGURE S2 Outcome based clustering analysis of CAPNETZ participants 00374-2025.SUPPLEMENT2 FIGURE S3 Frequencies and mortality rates following predictive SOM clustering 00374-2025.SUPPLEMENT3 FIGURE S4 Comparing mortality over 180 day follow up between high risk clusters for post acute mortality 00374-2025.SUPPLEMENT4 FIGURE S5 Predictive strength of baseline CRP for C.13 15 over 180 day follow up 00374-2025.SUPPLEMENT5 FIGURE S6 Predictive strength of baseline BMI for C.13 15 over 180 day follow up 00374-2025.SUPPLEMENT6 FIGURE S7 Predictive strength of baseline Urea for C.13 15 over 180 day follow up 00374-2025.SUPPLEMENT7
