# Supplementary material for: Self-organising map clustering identifies high-risk clusters of post-acute mortality in a prospective multicentre study of community-acquired pneumonia
Source: ERJ Open Res. 2026 Jan 19;12(1):00374-2025. doi: 10.1183/23120541.00374-2025 (PMC12813681; doi:10.1183/23120541.00374-2025)
Supplement: Supplementary file 1 [file 00374-2025.SUPPLEMENT.pdf]

Supplement to: Self-organising map clustering identifies high-risk clusters of post-acute mortality in a prospective multicenter study

Hendrik Pott[1,2], Swetlana Gaffron[3], Roman Martin[4], Dieter Maier[5], Max Kutzinski[1,2], Barbara Weckler[1,2], Wilhelm Bertrams[2], Anna Lena Jung[2], Katrin Laakmann[2], Dominik Heider[4], Claus F. Vogelmeier [6,8], Gernot Rohde[7,8], CAPNETZ Study Group, Bernd Schmeck[1,2,8,9, 10 $]

[1] Department of Medicine, Pulmonary and Critical Care Medicine, Clinic for Airway Infections, University Medical Centre Marburg, Philipps-University Marburg, Marburg, Germany.

[2] Institute for Lung Research, Universities of Giessen and Marburg Lung Centre, Philipps-University Marburg, Marburg, Germany.

[3] Viscovery Software GmbH, Vienna, Austria.

[4] Institute of Medical Informatics, University of Münster, Münster Germany.

[5] Labvantage-Biomax GmbH, München, Germany.

[6] Department of Medicine, Pulmonary and Critical Care Medicine, University Medical Centre Marburg, Philipps-University Marburg.

[7] Department of Respiratory Medicine, University Hospital Frankfurt, Frankfurt, Germany.

[8] Member of the German Centre for Lung Research (DZL), Germany.

[9] Member of the German Centre of Infectious Disease Research, Marburg, Germany.

[10] Institute for Lung Health, Giessen, Germany.

$ Corresponding author, Institute for Lung Research, BMFZ, Hans-Meerwein- Str. 2, 35043 Marburg, phone: +49 6421 28 65713, fax: +49 6421 28 21993, e-mail: bernd.schmeck@staff.uni-marburg.de

Figure Captions

Figure S1 Patient records available for our analysis.

Figure S2 Outcome-based clustering analysis of CAPNETZ participants. 7840 eligible CAPNETZ patients were arranged in a self-organising map (SOM). Similarity of patient data was encoded by proximity of placement on the map, with more diverse patients at higher distance to each other. Arithmetic mean of assessed values in each node was color-coded as indicated by the respective panel legends. Clustering was performed based on outcome, with 280 patients in the 30-day mortality cluster (D30), 315 patients in the 31-180-day mortality cluster (D31-180), 120 survivors who needed ICU admission in the ICU cluster, 893 survivors who needed change of the initially chosen antibiotic treatment in the change cluster, and 6232 in the rest cluster. Differential distribution of clinical attributes was inspected using the Viscovery software.

Figure S3 Frequencies and mortality rates following predictive SOM-clustering. Patient frequencies per cluster were represented by a bar plot, with an overlaying graph plot indicating % of acute (blue) and non-acute (red) mortality in that cluster.

Figure S4 Comparing mortality over 180 day follow-up between high-risk clusters for post-acute mortality. Kaplan-Meier models were applied and plotted using the lifelines package in python v.3.8.

Figure S5 Predictive strength of baseline CRP for C.13-15 over 180-day follow up. High non-acute mortality clusters C.13-15 were investigated for biomarkers predictive of non-acute mortality, predictive

strength was explored by calculation of cumulative/dynamic AUC. The mean AUC was indicated by dotted lines in the respective colour used to plot the model.

Figure S6 Predictive strength of baseline BMI for C.13-15 over 180-day follow up. High non-acute mortality clusters C.13-15 were investigated for biomarkers predictive of non-acute mortality, predictive strength was explored by calculation of cumulative/dynamic AUC. The mean AUC was indicated by dotted lines in the respective colour used to plot the model.

Figure S7 Predictive strength of baseline Urea for C.13-15 over 180-day follow up. High non-acute mortality clusters C.13-15 were investigated for biomarkers predictive of non-acute mortality, predictive strength was explored by calculation of cumulative/dynamic AUC. The mean AUC was indicated by dotted lines in the respective colour used to plot the model.

Supplemental Table 1: *Included vs excluded patients from cohort*

**Characteristic**	**Excluded**, N = 3992 (34%)[1]	**Included**, N = 7840 (66%)[1]	**p-value**[2]
Age	57.3 ± 19.3	62.3 ± 17.5	<0.001
BMI	25.3 ± 5.1	26.2 ± 5.7	<0.001
Urea [mmol/l]	6.3 ± 4.8	7.1 ± 5.4	<0.001
Hemoglobin	8.4 ± 1.5	8.2 ± 1.1	<0.001
CRP [mg/dl]	88.9 ± 100.0	137.0 ± 122.8	<0.001
O2 saturation [%]	93.2 ± 5.0	92.2 ± 5.9	<0.001
Female	2,064.0 (51.7%)	3,115.0 (39.7%)	<0.001
Dyspnoea	2,697.0 (68.4%)	5,819.0 (74.4%)	<0.001
Systolic blood pressure < 100 mmHg	490.0 (12.5%)	756.0 (9.7%)	<0.001
Oxygen application [0,1]	998.0 (25.1%)	4,774.0 (61.0%)	<0.001
Number of acute organ dysfunctions [0-9]	2.7 (1.7)	3.3 (1.5)	<0.001

[1]Mean ± SD; n (%); Mean (SD)

[2]Welch-ANOVA; One-way ANOVA; Pearson's Chi-squared test; Kruskal-Wallis rank sum test

Supplemental Table 2: *Clinical characteristics of outcome groups used for exploratory clustering*

Variable	**D30**, N = 280 (3.6%)[1]	**D31-180**, N = 315 (4.0%)[1]	**ICU**, N = 120 (1.5%)[1]	**Change**, N = 893 (11%)[1]	**Rest**, N = 6232 (79%)[1]	**p-value**[2]	D31-180 vs. ICU[3]	D31-180 vs. Change[3]	D31-180 vs. Rest[3]
Leukocytes [/nl]	14.9 ± 8.3	13.5 ± 6.4	14.4 ± 7.4	13.5 ± 6.8	12.9 ± 6.0	<0.001	0.7	>0.9	0.5
Platelets [k/µl]	275.6 ± 123.0	278.5 ± 124.2	238.8 ± 107.2	258.9 ± 117.6	259.3 ± 110.7	0.003	0.011	0.11	0.060
Female	83.0 (29.6%)	82.0 (26.0%)	39.0 (32.5%)	338.0 (37.8%)	2,573.0 (41.3%)	<0.001	>0.9	0.001	<0.001
Heart failure [0,1]	120.0 (52.2%)	117.0 (47.8%)	37.0 (46.3%)	148.0 (28.4%)	1,106.0 (31.7%)	<0.001	>0.9	<0.001	<0.001
COPD [0,1]	23.0 (12.2%)	34.0 (17.4%)	32.0 (29.6%)	62.0 (9.3%)	528.0 (11.4%)	<0.001	0.2	0.026	0.2
Smoker during the last 12 months	58.0 (22.7%)	77.0 (24.8%)	41.0 (35.7%)	243.0 (27.7%)	1,919.0 (31.0%)	0.002	0.3	>0.9	0.2
Chronic renal disease [0,1]	66.0 (28.7%)	64.0 (26.2%)	17.0 (21.3%)	80.0 (15.3%)	554.0 (15.9%)	<0.001	>0.9	0.005	<0.001
Cerebrovascular disease [0,1]	43.0 (18.8%)	52.0 (21.3%)	11.0 (13.8%)	80.0 (15.4%)	410.0 (11.7%)	<0.001	>0.9	0.5	<0.001
Number of comorbidities [0..9]	3.4 (1.6)	3.2 (1.5)	3.2 (1.9)	2.4 (1.5)	2.4 (1.5)	<0.001	>0.9	0.007	0.002

[1]Mean ± SD; n (%); Mean (SD)

[2]Welch-ANOVA; Pearson's Chi-squared test; Kruskal-Wallis rank sum test

[3]Post-hoc test p-values: Games-Howell test, Dunn's test,
Fisher's exact test.

Supplemental Table 3: *Attributes chosen for predictive clustering, with post-hoc tests.*

**Variable**	**D30**, N = 280 (3.6%)[1]	**D31-180**, N = 315 (4.0%)[1]	**ICU**, N = 120 (1.5%)[1]	**Change**, N = 893 (11%)[1]	**Rest**, N = 6232 (79%)[1]	**p-value**[2]	D31-180 vs. ICU[3]	D31-180 vs. Change[3]	D31-180 vs. Rest[3]

Variable	**D30**, N = 280 (3.6%)[1]	**D31-180**, N = 315 (4.0%)[1]	**ICU**, N = 120 (1.5%)[1]	**Change**, N = 893 (11%)[1]	**Rest**, N = 6232 (79%)[1]	**p-value**[2]	D31-180 vs. ICU[3]	D31-180 vs. Change[3]	D31-180 vs. Rest[3]
Age	73.86 ± 12.32	73.60 ± 11.12	63.63 ± 15.79	62.40 ± 17.11	61.20 ± 17.65	<0.001	<0.001	<0.001	<0.001
BMI	24.77 ± 6.17	25.20 ± 5.35	26.65 ± 5.97	25.94 ± 5.40	26.32 ± 5.66	<0.001	0.15	0.2	0.004
Systolic blood pressure < 100 mmHg	77.00 (27.80%)	38.00 (12.10%)	21.00 (17.50%)	92.00 (10.37%)	528.00 (8.52%)	<0.001	>0.9	>0.9	0.4
Dyspnoea	255.00 (93.07%)	258.00 (82.43%)	107.00 (89.17%)	652.00 (73.18%)	4,547.00 (73.09%)	<0.001	>0.9	0.010	0.002
Number of acute organ dysfunctions [0-9]	4.00 (3.00 - 5.00)	4.00 (3.00 - 5.00)	5.00 (4.00 - 6.00)	3.00 (2.00 - 4.00)	3.00 (2.00 - 4.00)	<0.001	<0.001	<0.001	<0.001
O2 saturation [%]	89.72 ± 6.83	89.98 ± 7.89	87.76 ± 9.65	92.70 ± 5.33	92.44 ± 5.65	<0.001	0.3	<0.001	<0.001
Oxygen application [0,1]	250.00 (89.61%)	241.00 (76.51%)	111.00 (92.50%)	580.00 (65.10%)	3,592.00 (57.71%)	<0.001	<0.001	0.002	<0.001
Hemoglobin	7.73 ± 1.40	7.78 ± 1.29	8.38 ± 1.38	8.20 ± 1.13	8.26 ± 1.11	<0.001	<0.001	<0.001	<0.001
CRP [mg/dl]	152.88 ± 122.14	110.48 ± 97.19	160.90 ± 158.25	157.74 ± 131.61	134.22 ± 121.40	<0.001	0.012	<0.001	<0.001
Urea [mmol/l]	11.97 ± 8.02	9.94 ± 8.19	9.98 ± 6.62	7.21 ± 5.26	6.66 ± 4.84	<0.001	>0.9	<0.001	<0.001

[1]Mean ± SD; n (%); Median (IQR)

[2]Welch-ANOVA; Pearson's Chi-squared test; Kruskal-Wallis rank sum test

[3]Post-hoc test p-values: Games-Howell test, Dunn's test,
Fisher's exact test.

Supplemental Table 4: *Constructed Random survival forest models*

Patient group	Number of patients	Train/Test split	Assessed Follow-up	Harrel's C/Uno's C
Whole cohort	7840	0.65/0.35	180 days	0.81 / 0.81
Prediction cohort	7309	0.65/0.35	180 days	0.83 / 0.83
Cluster 1-8	5494	0.65/0.35	180 days	0.77 / 0.77
Cluster 9-10	163	0.65/0.35	180 days	0.7 / 0.7
Cluster 11	773	0.65/0.35	180 days	0.58 / 0.58

Cluster 12 - 15	879	0.65/0.35	180 days	0.69 / 0.69
Cluster 13	167	0.65/0.35	180 days	0.84 / 0.84
Cluster 14	312	0.65/0.35	180 days	0.65 / 0.65
Cluster 15	359	0.65/0.35	180 days	0.60 / 0.60
Whole cohort	7559	0.65/0.35	31 - 180 days	0.79 / 0.79
Prediction cohort	7053	0.65/0.35	31 - 180 days	0.76 / 0.76
Cluster 1-8	5393	0.65/0.35	31 - 180 days	0.77 / 0.77
Cluster 9-10	128	0.65/0.35	31 - 180 days	0.44 / 0.44
Cluster 11	739	0.65/0.35	31 - 180 days	0.48 / 0.48
Cluster 12 - 15	793	0.65/0.35	31 - 180 days	0.66 / 0.66
Cluster 13	141	0.65/0.35	31 - 180 days	0.71 / 0.71
Cluster 14	284	0.65/0.35	31 - 180 days	0.91 / 0.91
Cluster 15	331	0.65/0.35	31 - 180 days	0.53 / 0.53

Supplemental Table 5: *Feature importances of study cohort and selected cohort*

**Whole cohort,** 180-day Follow-up, N = 7840		**Whole cohort,** 31-180-day Follow-up, N = 7559		**Clustering cohort,** 180-day Follow-up, N = 7309		**Clustering cohort,** 31-180-day Follow-up, N = 7053	
**Feature**	**Permutation-based feature importance**[1]	**Feature**	**Permutation-based feature importance**[1]	**Feature**	**Permutation-based feature importance**[1]	**Feature**	**Permutation-based feature importance**[1]
Age	0.06 ± 0.01	Age	0.04 ± 0.01	Age	0.07 ± 0.01	Age	0.06 ± 0.01
Urea	0.01 ± 0.01	Urea	0.01 ± 0.01	ICU 1 incl ventilation or catecholamines	0.02 ± 0.0	crb65	0.02 ± 0.01
Tumor 0	0.01 ± 0.0	crb65	0.01 ± 0.01	Urea	0.02 ± 0.0	Fever R	0.02 ± 0.01
Hemoglobin	0.01 ± 0.0	Hemoglobin	0.01 ± 0.01	BMI	0.02 ± 0.0	Platelets	0.01 ± 0.0
BMI	0.01 ± 0.0	Fever R	0.01 ± 0.0	O2 saturation	0.01 ± 0.0	Hemoglobin	0.01 ± 0.01
ICU 1 incl ventilation or catecholamines	0.01 ± 0.0	BMI	0.01 ± 0.0	crb65	0.01 ± 0.0	O2 saturation	0.01 ± 0.0
crb65	0.0 ± 0.0	Platelets	0.0 ± 0.01	Tumor 0	0.01 ± 0.0	Urea	0.0 ± 0.0
O2 saturation	0.0 ± 0.0	Female	0.0 ± 0.0	Fever R	0.01 ± 0.0	Tumor 0	0.0 ± 0.0
Fever R	0.0 ± 0.0	Tumor 0	0.0 ± 0.0	Platelets	0.01 ± 0.0	SOFA Coagulation	0.0 ± 0.0
Chronic respiratory disease	0.0 ± 0.0	Chronic respiratory disease	0.0 ± 0.0	Hemoglobin	0.0 ± 0.0	Low mean blood pressure 0	0.0 ± 0.0

[1]mean ± standard deviation, in descending order of mean.

Supplemental Table 6: *Logranktest comparing cluster groups*

Group A	Group B	P
C. 1-8	C. 11	< 0.005
C. 1-8	C. 12-15	< 0.005
C. 1-8	C. 9-10	< 0.005
C. 11	C. 12-15	< 0.005
C. 11	C. 9-10	< 0.005
C. 12-15	C. 9-10	0.03

Supplemental Table 7: *Comparing C9-10 vs C12-15 at specific time points*

Days	P
30	< 0.001
60	0.002
90	0.023
120	0.022
150	0.048
160	0.063
180	0.071

Supplemental Table 8: *Comparing clusters with high non-acute mortality*

Group A	Group B	P
11	12	0.1
11	13	< 0.005
11	14	< 0.005
11	15	< 0.005
12	13	0.37
12	14	0.83
12	15	0.99
13	14	0.17
13	15	0.07
14	15	0.67

Supplemental Table 9: *Feature importances of Cluster groups C9-10 and C12-15*

**C9-10,** 180-day Follow-up, N = 163		**C9-10,** 31-180-day Follow-up, N = 128		**C12 - 15,** 180-day Follow-up, N = 879		**C12 - 15,** 31-180-day Follow-up, N = 793	
**Feature**	**Permutation-based feature importance[1]**	**Feature**	**Permutation-based feature importance[1]**	**Feature**	**Permutation-based feature importance[1]**	**Feature**	**Permutation-based feature importance[1]**
Tumor 0	0.13 ± 0.15	Tumor 0	0.12 ± 0.03	BMI	0.11 ± 0.02	BMI	0.1 ± 0.04
Hemoglobin	0.07 ± 0.02	Hemoglobin	0.09 ± 0.07	Number symptoms R	0.02 ± 0.01	Vaccination Influenza H	0.02 ± 0.01
BP diastolic 0	0.02 ± 0.02	Expectorants	0.02 ± 0.02	Fever R	0.02 ± 0.01	Age	0.02 ± 0.01
Breathing non-invasively	0.02 ± 0.02	BP systolic 0	0.01 ± 0.01	CRP	0.01 ± 0.01	CRP	0.01 ± 0.01
Female	0.01 ± 0.02	Female	0.01 ± 0.01	BP systolic 0	0.01 ± 0.0	Chronic respiratory disease	0.01 ± 0.01
Sputum R	0.01 ± 0.01	Fever 0	0.01 ± 0.01	Chronic respiratory disease	0.01 ± 0.01	Number symptoms R	0.01 ± 0.02
BP systolic 0	0.01 ± 0.05	High heart rate 0	0.01 ± 0.01	Low mean blood pressure 0	0.0 ± 0.0	Smoker last 12 month 0	0.01 ± 0.0
Number symptoms R	0.01 ± 0.01	Vaccination Influenza H	0.01 ± 0.02	Age	0.0 ± 0.01	Urea	0.01 ± 0.0
Platelets	0.01 ± 0.02	SOFA Coagulation	0.01 ± 0.01	ICU 1 incl ventilation or catecholamines	0.0 ± 0.0	Fever R	0.01 ± 0.02
Vaccination Influenza H	0.01 ± 0.01	O2 saturation	0.01 ± 0.02	Vaccination Influenza H	0.0 ± 0.0	Female	0.0 ± 0.0

[1]mean ± standard deviation, in descending order of mean.

Supplemental Table 10: *Feature importances of Cluster groups C1-8 and C11*

**C1-8,** 180-day Follow-up, N = 5494	**C1-8,** 31-180-day Follow-up, N = 5393	**C11,** 180-day Follow-up, N = 773	**C11,** 31-180-day Follow-up, N = 739

Feature	Permutation-based feature importance[1]	Feature	Permutation-based feature importance[1]	Feature	Permutation-based feature importance[1]	Feature	Permutation-based feature importance[1]
Age	0.03 ± 0.01	Age	0.04 ± 0.03	Age	0.04 ± 0.01	BMI	0.03 ± 0.05
Tumor 0	0.03 ± 0.01	Tumor 0	0.01 ± 0.01	BP systolic 0	0.03 ± 0.01	Positive auscultation result R	0.03 ± 0.01
Chronic respiratory disease 0 incl. COPD, LTOT, CPAP	0.02 ± 0.01	Chronic respiratory disease 0 incl. COPD, LTOT, CPAP	0.01 ± 0.0	O2 saturation	0.02 ± 0.03	CRP	0.02 ± 0.01
Urea	0.02 ± 0.01	Female	0.01 ± 0.0	crb65	0.01 ± 0.01	Age	0.02 ± 0.01
ICU 1 incl ventilation or catecholamines	0.01 ± 0.01	Chronic respiratory disease	0.01 ± 0.01	Vaccination Influenza H	0.01 ± 0.0	Urea	0.02 ± 0.0
BP diastolic 0	0.01 ± 0.0	crb65	0.01 ± 0.02	Smoker last 12 month 0	0.01 ± 0.01	Respiratory rate 0	0.01 ± 0.01
O2 saturation	0.01 ± 0.0	Fever R	0.01 ± 0.01	Sputum R	0.01 ± 0.01	crb65	0.01 ± 0.01
CRP	0.01 ± 0.0	O2 saturation	0.0 ± 0.0	LTOT 0	0.01 ± 0.01	LTOT 0	0.01 ± 0.0
Sodium	0.01 ± 0.0	Chronic respiratory disease 0 incl. COPD 0	0.0 ± 0.0	Pleural effusion 0	0.01 ± 0.02	SOFA Coagulation	0.01 ± 0.01
Platelets	0.01 ± 0.0	BP systolic 0	0.0 ± 0.0	Heart rate 0	0.0 ± 0.01	qSOFA	0.01 ± 0.0

[1]mean ± standard deviation, in descending order of mean.

Supplemental Table 11: *Baseline attributes of high non-acute mortality clusters*

Characteristic	13, N = 167[1]	14, N = 312[1]	15, N = 359[1]	p-value[2]
Age	72 ± 11	77 ± 8	78 ± 8	<0.001
Female	48 (29%)	110 (35%)	116 (32%)	0.3
BMI	25.6 ± 7.3	25.9 ± 5.1	26.8 ± 5.3	0.056
Hemoglobin [mmol/l]	7.85 ± 1.46	8.08 ± 1.24	7.06 ± 0.91	<0.001
Leukocytes [k/µl]	16.2 ± 9.3	14.3 ± 6.3	12.4 ± 4.7	<0.001
Platelets [k/µl]	241 ± 117	242 ± 111	245 ± 107	0.9
Urea [mmol/l]	16 ± 8	10 ± 6	16 ± 7	<0.001
Heart failure [0,1]	59 (41%)	134 (50%)	171 (53%)	0.062
Chronic renal disease [0,1]	51 (36%)	55 (20%)	144 (45%)	<0.001
Diabetes [0,1]	30 (21%)	97(36%)	135(42%)	<0.001
Cerebrovascular disease [0,1]	19 (13%)	95 (35%)	49 (15%)	<0.001

[1]Mean ± SD; n (%); Median (IQR)

[2]Welch-ANOVA; Pearson's Chi-squared test; One-way ANOVA

Supplemental Table 12: *Feature importances of C13-C14*

**C13,** 180-day Follow-up, N = 167		**C13,** 31-180-day Follow-up, N = 141		**C14,** 180-day Follow-up, N = 312		**C14,** 31-180-day Follow-up, N = 284	
**Feature**	**Permutation-based feature importance[1]**	**Feature**	**Permutation-based feature importance[1]**	**Feature**	**Permutation-based feature importance[1]**	**Feature**	**Permutation-based feature importance[1]**
Age	0.06 ± 0.03	BMI	0.04 ± 0.09	BMI	0.11 ± 0.02	Heart rate 0	0.09 ± 0.05
CRP	0.05 ± 0.03	CRP	0.0 ± 0.02	Platelets	0.06 ± 0.04	BMI	0.07 ± 0.02
BMI	0.03 ± 0.03	Leukocytes, /nL	0.0 ± 0.03	Age	0.04 ± 0.02	Platelets	0.07 ± 0.03
Urea	0.02 ± 0.02	Sputum R	0.0 ± 0.0	Leukocytes, /nL	0.03 ± 0.03	Sodium	0.04 ± 0.02
Sodium	0.02 ± 0.01	Positive auscultation result R	0.0 ± 0.0	BP systolic 0	0.03 ± 0.03	Age	0.04 ± 0.01
Platelets	0.01 ± 0.01	Infiltrate R	0.0 ± 0.0	Urea	0.02 ± 0.02	Fever 0	0.03 ± 0.02
Pleural effusion 0	0.01 ± 0.02	ICU 1 incl ventilation or catecholamines	0.0 ± 0.0	O2 saturation	0.02 ± 0.02	crb65	0.03 ± 0.02
Respiratory rate 0	0.01 ± 0.01	CPAP mask 0	0.0 ± 0.0	Fever R	0.02 ± 0.01	CRP	0.03 ± 0.01
O2 saturation	0.01 ± 0.01	CPAP tracheo 0	0.0 ± 0.0	Tumor 0	0.01 ± 0.02	Fever R	0.02 ± 0.01
Heart rate 0	0.0 ± 0.01	Chronic respiratory disease 0 incl. COPD 0	0.0 ± 0.0	High blood pressure 0	0.01 ± 0.01	BP systolic 0	0.02 ± 0.05

[1]mean ± standard deviation, in descending order of mean.

Supplemental Table 13: *Feature importances of C15*

**C15,** 180-day Follow-up, N = 359		**C15,** 31-180-day Follow-up, N = 331	
**Feature**	**Permutation-based feature importance**[1]	**Feature**	**Permutation-based feature importance**[1]
BMI	0.11 ± 0.02	BMI	0.1 ± 0.04
Number symptoms R	0.02 ± 0.01	Vaccination Influenza H	0.02 ± 0.01
Fever R	0.02 ± 0.01	Age	0.02 ± 0.01
CRP	0.01 ± 0.01	CRP	0.01 ± 0.01
BP systolic 0	0.01 ± 0.0	Chronic respiratory disease	0.01 ± 0.01
Chronic respiratory disease	0.01 ± 0.01	Number symptoms R	0.01 ± 0.02
Low mean blood pressure 0	0.0 ± 0.0	Smoker last 12 month 0	0.01 ± 0.0
Age	0.0 ± 0.01	Urea	0.01 ± 0.0
ICU 1 incl ventilation or catecholamines	0.0 ± 0.0	Fever R	0.01 ± 0.02
Vaccination Influenza H	0.0 ± 0.0	Female	0.0 ± 0.0

[1]mean ± standard deviation, in descending order of mean.

Supplemental Table 14: *Modelling 31-180-day mortality including interactions between baseline CRP and C13-15*

Feature	Hazard Ratio [95 % CI]	P
Female	0.58 [0.44 - 0.75]	< 0.001
Age[1]	1.05 [1.04 - 1.06]	< 0.001
Smoker last 12 month 0	1.49 [1.12 - 1.99]	0.01
CRP[1,2]	0.95 [0.91 - 1.0]	0.06
Is_C13	2.8 [1.61 - 4.85]	< 0.001
C13_x_CRP[1,2]	0.79 [0.65 - 0.96]	0.02
Is_C14	2.42 [1.66 - 3.51]	< 0.001
C14_x_CRP[1,2]	1.03 [0.9 - 1.17]	0.7
Is_C15	3.4 [2.07 - 5.58]	< 0.001
C15_x_CRP[1,2]	1.27 [1.01 - 1.58]	0.04

[1]centered on the mean. [2]Steps of 40 mg/dl

Description: Results of cox proportional hazard model for 31-180-day follow-up of 6909 CAPNETZ participants, comparing CRP-related hazard for patients in cluster 13, 14 and 15.

Supplemental Table 15: *Modelling 31-180-day mortality including interactions between baseline heart rate and C13-15*

Feature	Hazard Ratio [95 % CI]	P

Feature	Hazard Ratio [95 % CI]	P
Female	0.57 [0.44 - 0.75]	< 0.001
Age[1]	1.05 [1.04 - 1.06]	< 0.001
Smoker last 12 month	1.4 [1.05 - 1.86]	0.02
Heart rate[1]	1.01 [1.0 - 1.01]	0.04
Is_C13	12.64 [1.43 - 111.46]	0.02
C13_x_Heart rate[1]	0.98 [0.96 - 1.01]	0.16
Is_C14	1.89 [0.3 - 11.9]	0.5
C14_x_Heart rate[1]	1.0 [0.98 - 1.02]	0.81
Is_C15	1.25 [0.27 - 5.74]	0.77
C15_x_Heart rate[1]	1.01 [0.99 - 1.02]	0.37

[1]centered on the mean.

Description: Results of cox proportional hazard model for 31-180-day follow-up of 6980 CAPNETZ participants, comparing Heart rate-related hazard for patients in cluster 13, 14 and 15.

Supplemental Table 16: *Modelling 31-180-day mortality including interactions between baseline BMI and C13-15*

Feature	Hazard Ratio [95 % CI]	P
Female	0.55 [0.42 - 0.72]	< 0.001
Age[1]	1.05 [1.04 - 1.06]	< 0.001
Smoker last 12 month 0	1.31 [0.98 - 1.76]	0.07
BMI[1]	0.98 [0.95 - 1.01]	0.24
Is_C13	1.82 [0.91 - 3.68]	0.09
C13_x_BMI[1]	0.89 [0.78 - 1.01]	0.07
Is_C14	1.93 [1.22 - 3.05]	0.005
C14_x_BMI[1]	0.88 [0.8 - 0.97]	0.01
Is_C15	2.37 [1.67 - 3.38]	< 0.001

Feature	Hazard Ratio [95 % CI]	P
C15_x_BMI[1]	0.97 [0.9 - 1.04]	0.42

[1]centered on the mean.

Description: Results of cox proportional hazard model for 31-180-day follow-up of 6939 CAPNETZ participants, comparing BMI-related hazard for patients in cluster 13, 14 and 15.

Supplemental Table 17: *Modelling 31-180-day mortality including interactions between baseline platelet concentration and C13-15*

Feature	Hazard Ratio [95 % CI]	P
Female	0.77 [0.66 - 0.91]	0.002
Age[1]	1.02 [1.01 - 1.02]	< 0.001
Smoker last 12 month 0	0.98 [0.82 - 1.17]	0.85
platelets[1,2]	1.03 [1.0 - 1.06]	0.03
Is_C13	2.12 [1.34 - 3.36]	0.001
C13_x_platelets[1,2]	1.02 [0.88 - 1.18]	0.79
Is_C14	2.05 [1.46 - 2.87]	< 0.001
C14_x_ platelets[1,2]	1.11 [1.03 - 1.21]	0.01
Is_C15	2.18 [1.61 - 2.95]	< 0.001
C15_x_platelets[1,2]	0.98 [0.88 - 1.09]	0.73

[1]centered on the mean. [2]increments of 40 platelets/nl

Description: Results of cox proportional hazard model for 31-180-day follow-up of 6980 CAPNETZ participants, comparing platelet-related hazard for patients in cluster 13, 14 and 15.

Supplemental Table 18: *Modelling 31-180-day mortality including interactions between baseline age and C13-15*

Feature	Hazard Ratio [95 % CI]	P
Female	0.58 [0.45 - 0.76]	< 0.001
Smoker last 12 month	1.43 [1.07 - 1.89]	0.01
Age[1]	1.06 [1.04 - 1.07]	< 0.001
Is_C13	3.45 [1.57 - 7.59]	0.002
C13_x_Age[1]	0.98 [0.93 - 1.03]	0.44
Is_C14	2.08 [0.86 - 5.07]	0.11
C14_x_ Age[1]	1.01 [0.96 - 1.06]	0.74
Is_C15	4.9 [2.36 - 10.16]	< 0.001
C15_x_ Age[1]	0.96 [0.92 - 1.0]	0.04

[1]centered on the mean.

Description: Results of cox proportional hazard model for 31-180-day follow-up of 6980 CAPNETZ participants, comparing age-related hazard for patients in cluster 13, 14 and 15.

Supplemental Table 19: *Modelling 31-180-day mortality including interactions between baseline urea and C13-15*

Feature	Hazard Ratio [95 % CI]	P
Female	0.59 [0.46 - 0.77]	< 0.001
Age[1]	1.05 [1.04 - 1.06]	< 0.001
Smoker last 12 month 0	1.34 [1.0 - 1.79]	0.05
Urea[1]	1.01 [0.98 - 1.04]	0.68
Is_C13	2.23 [1.07 - 4.64]	0.03
C13_x_urea[1]	1.02 [0.95 - 1.09]	0.6
Is_C14	2.35 [1.56 - 3.54]	< 0.001
C14_x_urea[1]	1.02 [0.96 - 1.07]	0.61
Is_C15	1.33 [0.79 - 2.22]	0.28
C15_x_urea[1]	1.06 [1.01 - 1.11]	0.02

[1]centered on the mean.

Description: Results of cox proportional hazard model for 31-180-day follow-up of 6521 CAPNETZ participants, comparing urea-related hazard for patients in cluster 13, 14 and
