# Supplementary figures and images for: Self-organising map clustering identifies high-risk clusters of post-acute mortality in a prospective multicentre study of community-acquired pneumonia
Source: ERJ Open Res. 2026 Jan 19;12(1):00374-2025. doi: 10.1183/23120541.00374-2025 (PMC12813681; doi:10.1183/23120541.00374-2025)

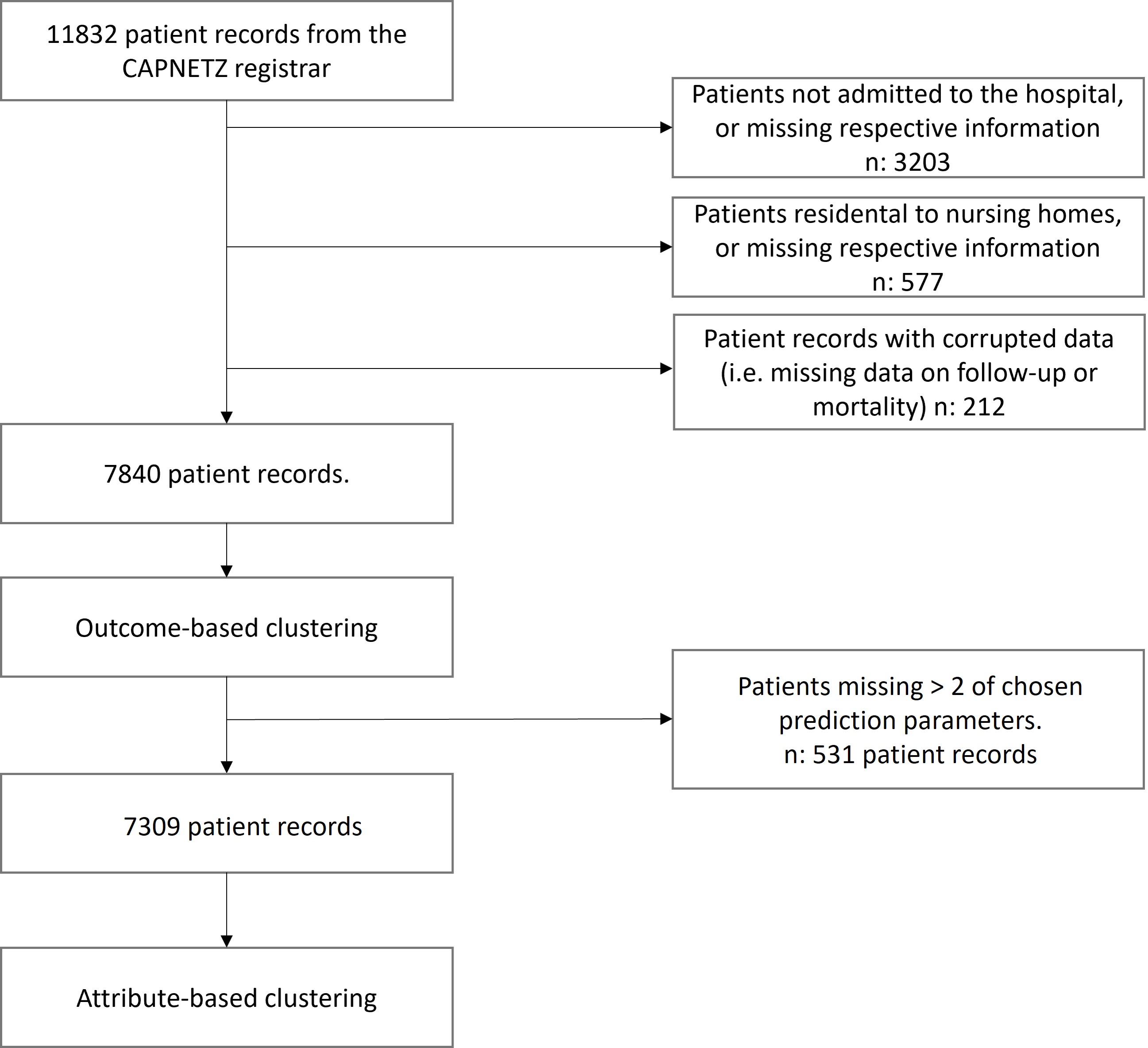

Supplement: Supplementary file 2 [file 00374-2025.SUPPLEMENT.png]

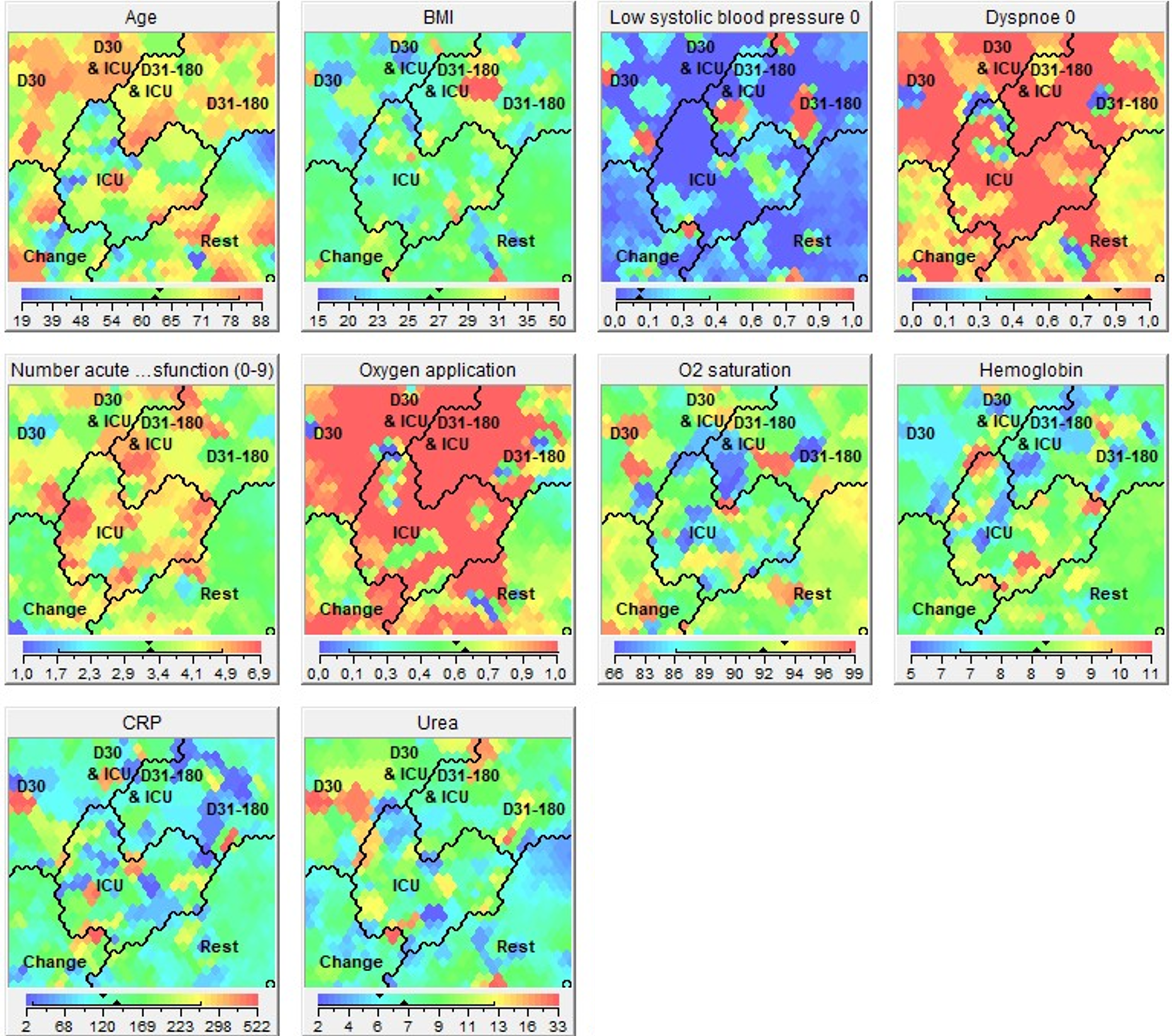

Supplement: Supplementary file 3 [file 00374-2025.SUPPLEMENT2.png]

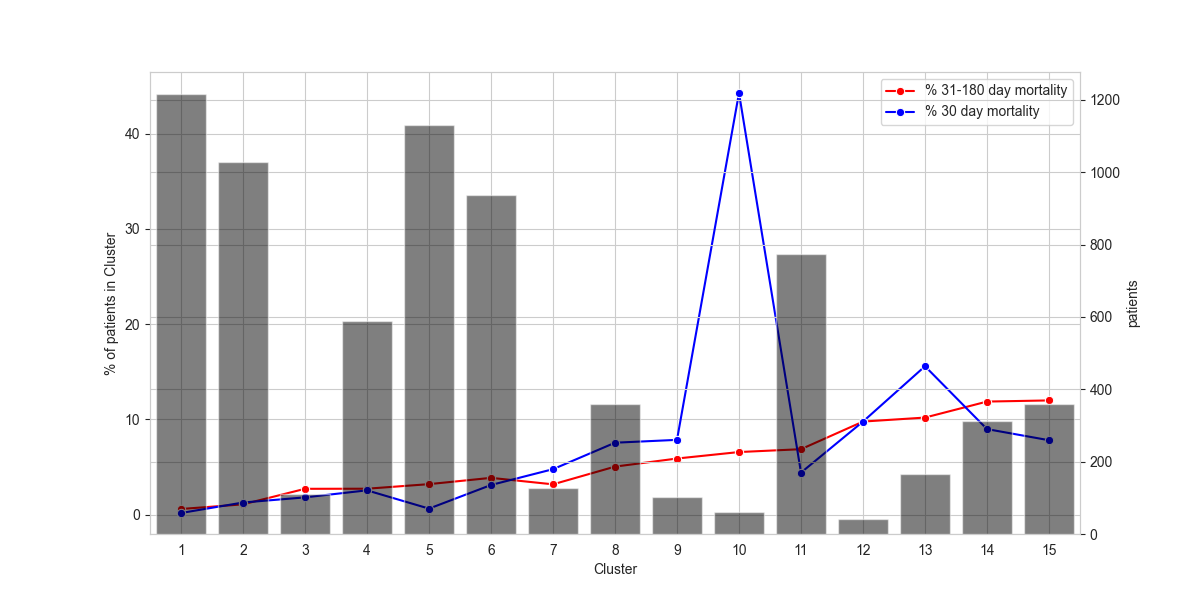

Supplement: Supplementary file 4 [file 00374-2025.SUPPLEMENT3.png]

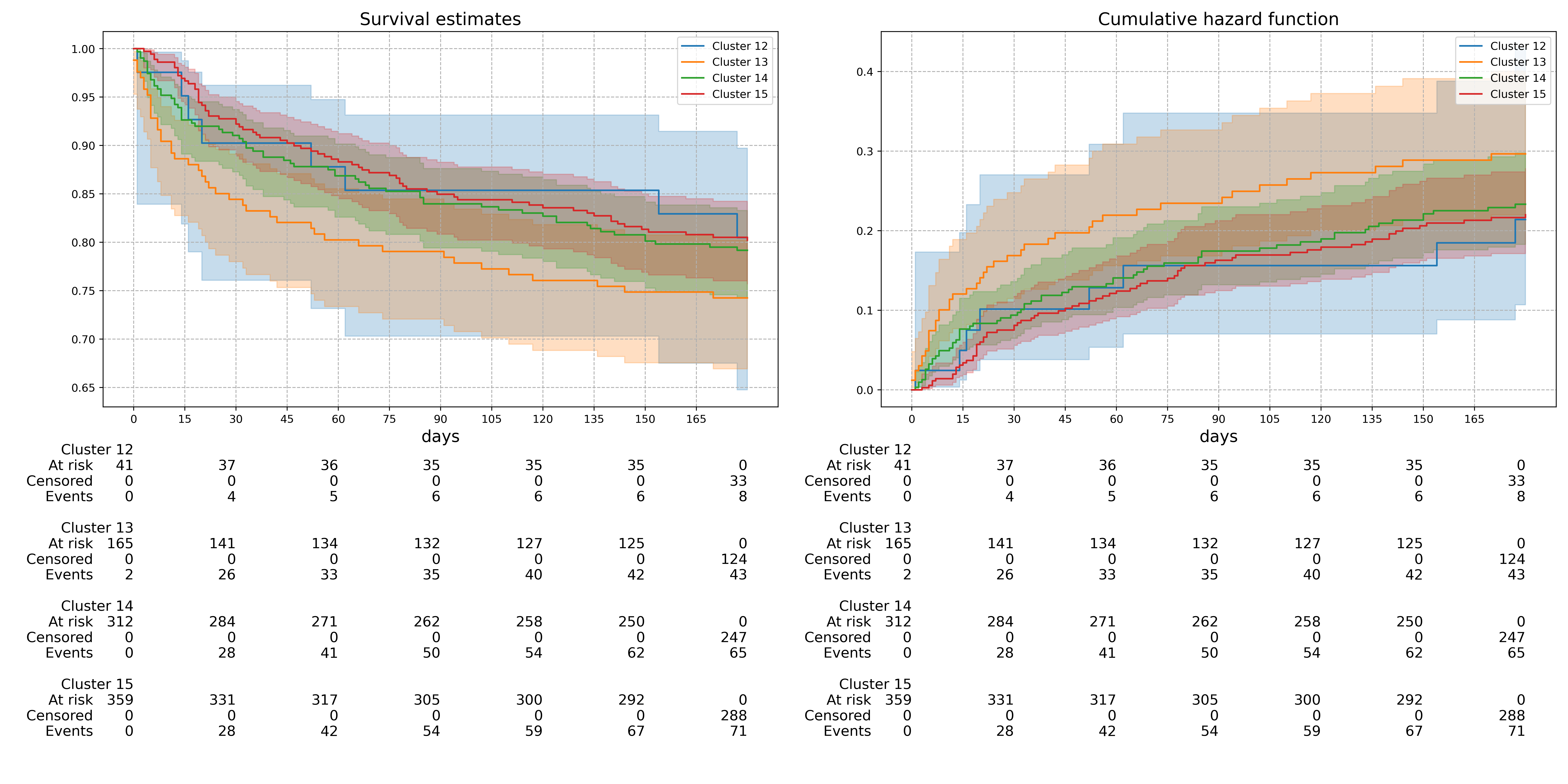

Supplement: Supplementary file 5 [file 00374-2025.SUPPLEMENT4.png]

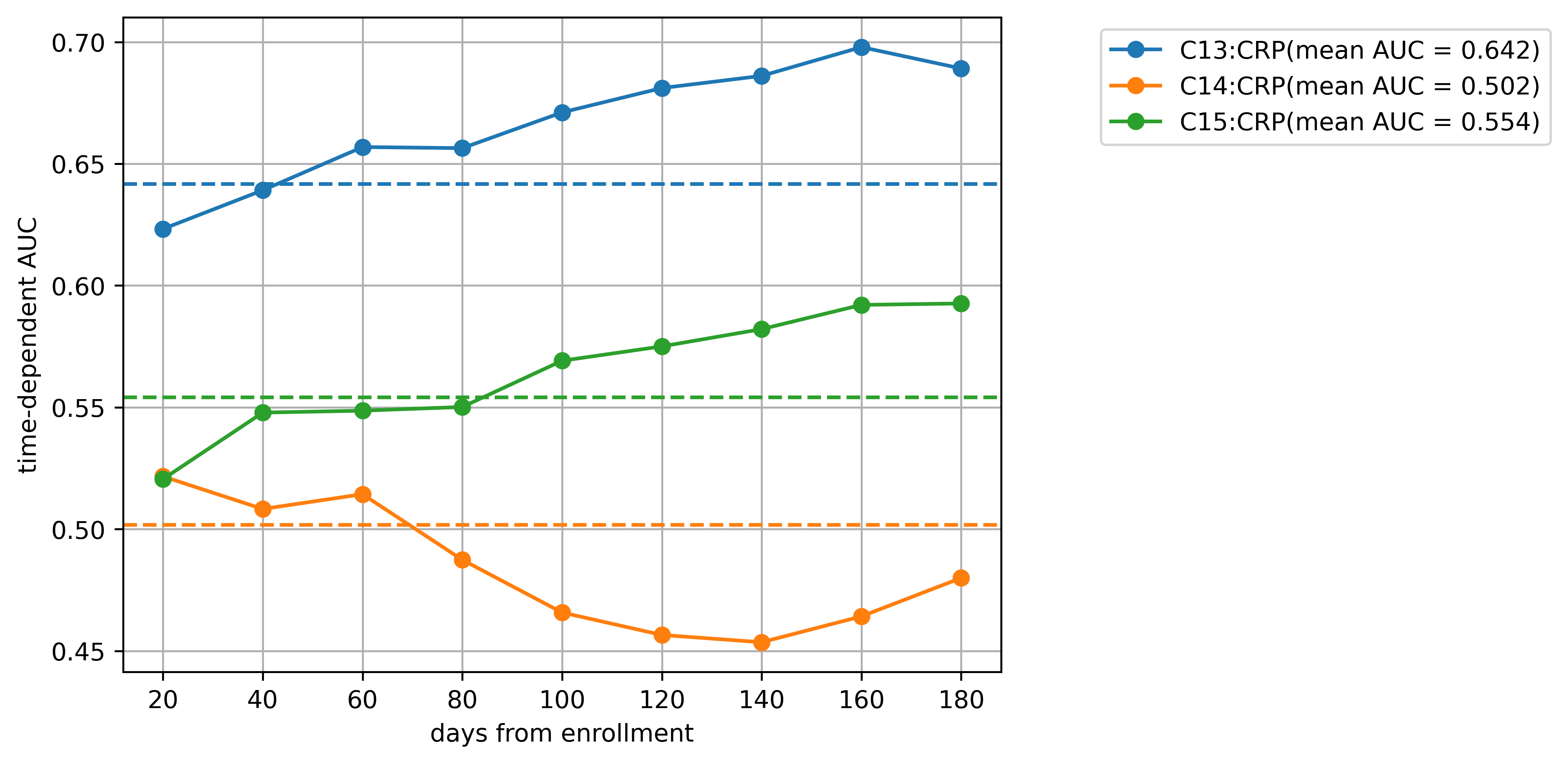

Supplement: Supplementary file 6 [file 00374-2025.SUPPLEMENT5.png]

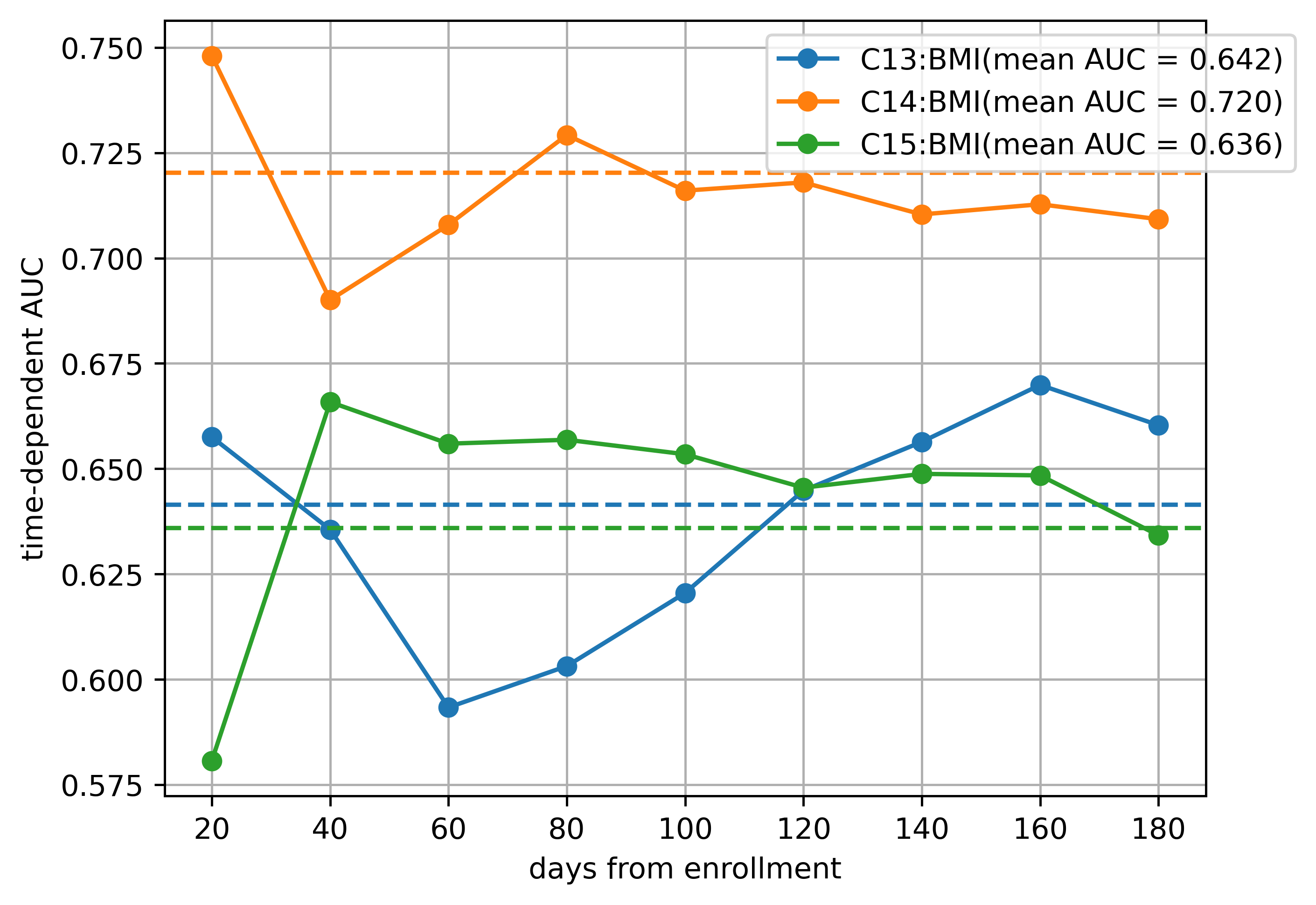

Supplement: Supplementary file 7 [file 00374-2025.SUPPLEMENT6.png]

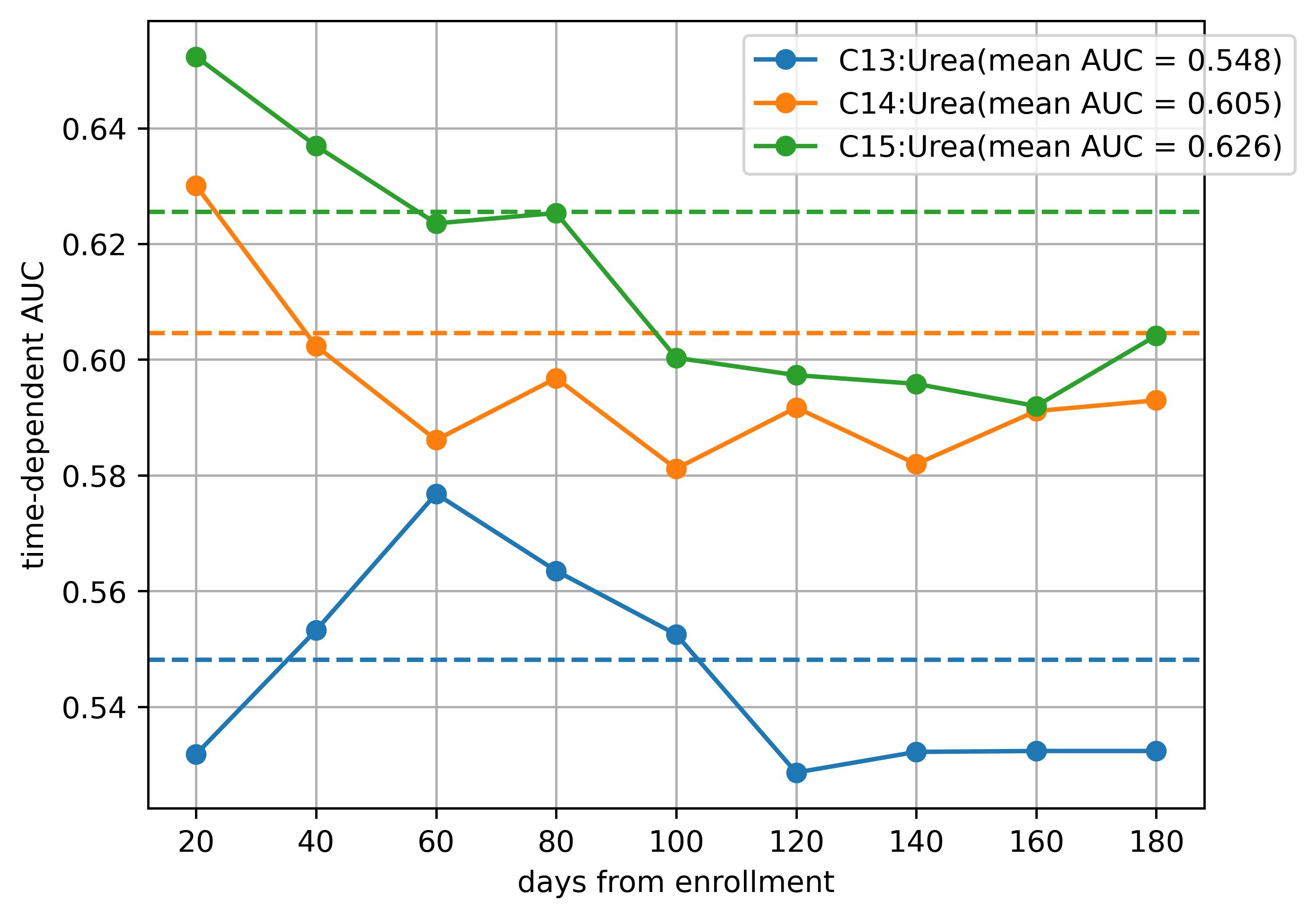

Supplement: Supplementary file 8 [file 00374-2025.SUPPLEMENT7.png]
